# Adoption, acceptability and sustained use of digital interventions to promote physical activity among inactive adults: a mixed-method study

**DOI:** 10.3389/fpubh.2023.1297844

**Published:** 2024-01-04

**Authors:** Unn S. Manskow, Edvard H. Sagelv, Konstantinos Antypas, Paolo Zanaboni

**Affiliations:** ^1^Norwegian Centre for E-Health Research, University Hospital of North Norway, Tromsø, Norway; ^2^Department of Health and Care Sciences, Faculty of Health Sciences, The Arctic University of Norway UiT, Tromsø, Norway; ^3^School of Sport Sciences, Faculty of Health Sciences, UiT The Arctic University of Norway, Tromsø, Norway; ^4^SINTEF Digital, Oslo, Norway; ^5^Department of Clinical Medicine, Faculty of Health Sciences, UiT The Arctic University of Norway, Tromsø, Norway

**Keywords:** digital intervention, RCT, physical activity, adoption, acceptability, sustained use, motivation, behavioral change

## Abstract

**Introduction:**

Despite the positive effects of physical activity (PA) to prevent lifestyle diseases and improve health and well-being, only one-third of Norwegian adults meet the minimum recommendations on PA. Digital interventions to promote PA in inactive adults may improve health and well-being by being available, personalized and adequate. Knowledge on users’ adoption, acceptability and sustainability of digital interventions to promote PA is still limited.

**Objective:**

To investigate the adoption, acceptability and sustained use of three digital interventions for promoting PA among inactive adults.

**Design:**

A randomized control trial (ONWARDS) with 183 participants assigned to 3 groups and followed up for 18 months. All participants received a wearable activity tracker with the personalized metric Personal Activity Intelligence (PAI) on a mobile app, two groups received additional access to online training and one group had also access to online social support.

**Methods:**

A mixed-methods approach was used to address the study objective. Acceptability was evaluated through the System Usability Scale (SUS) (*n* = 134) at 6 months. Adoption and sustained use were evaluated through a set of questions administered at 12 months (*n* = 109). Individual interviews were performed at 6 months with a sample of participants (*n* = 18). Quantitative data were analyzed with descriptive statistics, whereas qualitative data were analyzed using the Framework approach.

**Results:**

PAI was the most successful intervention, with satisfactory usability and positive effects on motivation and behavior change, contributing to high adoption and sustained use. Online social support had a high acceptability and sustained use, but the intervention was not perceived as motivational to increase PA. Online training had low adoption, usability and sustained use. The qualitative interviews identified five main themes: (1) overall approach to physical activity, (2) motivation, (3) barriers to perform PA, (4) effects of PA, and (5) usability and acceptability of the digital interventions.

**Conclusion:**

Personalized digital interventions integrating behavior change techniques such as individual feedback and goal setting are more likely to increase acceptability, adoption and sustained use. Future studies should investigate which digital interventions or combinations of different interventions are more successful in promoting PA among inactive adults according to the characteristics and preferences of the users.

**Trial registration:**

Clinical trial registered at ClinicalTrials.gov: NCT04526444.

## Introduction

Insufficient physical activity (PA) has major implications for health and the prevalence of lifestyle diseases like cardiovascular conditions, cancer and diabetes (1, 2). Active individuals have lower rates of all-cause mortality, lower risk to develop lifestyle diseases, higher level of cardiorespiratory and muscular fitness and healthier body mass and composition (3–5). In Norway, only one third of adults meets the minimum recommendation on PA, and in Northern Norway the PA levels are lower than for southern part of Norway (6).

Digital technology is increasingly used in interventions targeting PA, and some interventions show a potential to make PA more accessible to all age groups and support long-term adherence to PA recommendations (7, 8). Wearable activity trackers linked to mobile apps are now widely adopted and can assist individuals to be physically active (9, 10). Online home-based exercise programs have been reported to be feasible and effective in promoting PA in low-active older adults (10, 11). Peer support groups through social media has been shown to support adherence to PA and maintenance of behavioral change (12–15).

It is important to understand how digital interventions can address key barriers to perform PA in inactive adults, such as lack of motivation, lack of knowledge on how they can increase their level of PA, lack of facilities, weather conditions, time constraints and lack of social support (8, 16). Interventions based on behavior change techniques, including goal setting, monitoring, feedback, and social support, have shown to be effective at increasing PA levels among young adults in the shorter term (17). Digital interventions for PA based on wearable technology, SMS and mobile apps have the potential to promote health and well-being by improving availability, personalization and adequacy (18). However, while some studies have shown positive effects of digital interventions to increase PA levels in inactive adults, others have shown no effect at all (18–22).

The social cognitive theory is a well-known framework for behavior change, including the following five constructs: measurable outcomes (i.e., number of steps), proximal goal setting, procedural knowledge, perceived self-efficacy and the influence of social support (23, 24). Perceived self-efficacy, or beliefs about a person’s ability to carry out desired behaviors, affects both motivation and actions and is important for the sustainability in being physically active (25). Behavior change techniques such as goal setting, feedback, rewards and social factors are often included in digital interventions to promote physical activity, but it is unclear which of these components are most used and effective, and their effects on long-term adherence to PA (8, 17).

Implementation outcomes serve as necessary preconditions to understand if a digital intervention has obtained the desired effect (26). Acceptability is the perception of how a given treatment, service, practice or innovation is satisfactory, and low acceptability represents a well-known challenge in implementation research (26). Adoption is defined as the intention or action for the uptake or use of the intervention, whereas sustainability is described as maintenance, routinization and/or continuation of an intervention (26). A randomized controlled trial (RCT) on the acceptability and satisfaction of a web-based video-tailored PA intervention showed that the participants experienced the intervention insufficient to ensure good usage, perceived usefulness and satisfaction (27).

Despite the increasing number of studies on how digital interventions can promote PA, most interventions have a short-term duration (a few weeks or months), and the long-term effects are therefore unclear. In addition, most studies focus on specific age groups (e.g., young healthy individuals or older adults) or people from a specific disease group (e.g., cancer), measuring quantitative outcomes (15, 28–30). There is little knowledge on how digital interventions aimed to increase PA levels are accepted and used by inactive adults at risk to develop lifestyle diseases, and how they affect their motivation and perceived benefits (22). In addition, it is necessary to understand what influences people’s choice to take an intervention into use (i.e., adoption) and maintaining it in a long-term (sustained use).

We conducted a RCT aimed at evaluating the effects of three different digital health interventions on PA among adults recruited from the general population who were at risk of developing lifestyle diseases due to insufficient levels of PA (31). The purpose of this mixed-method study was to investigate inactive adults’ adoption, acceptability and sustained use of the digital interventions.

## Materials and methods

### Study setting

We conducted a hybrid type 1 effectiveness-implementation RCT aimed at an inactive and presumably high-risk population living in Northern Norway – the ONWARDS study (31). To be eligible for enrolment, participants had to fulfill the following inclusion criteria: (1) young (18–40 years) or middle aged (40–55 years) healthy adults, both men and women; (2) inactive (undertaking <150 min of moderate-intensity PA per week) in the last 3 months; (3) living in the Troms and Finnmark county; (4) current owner of a smartphone; and (5) able to understand training instructions in English language.

One hundred and eighty-three participants were randomized to 3 groups and participated for 18 months. The three groups were provided the following interventions: group (A) activity tracker (Mi Smart Band 5, Xiaomi, China) with the personalized metric Personal Activity Intelligence (PAI) on the mobile app PAI Health (PAI Health, Canada), group (B) activity tracker with PAI and access to online training videos (Les Mills+) to perform home-based training, group (C) activity tracker PAI, home-based online training and additional online social support via social media (Facebook). No control group receiving “standard care” was applied in this RCT, as inactive adults do not often access a training facility or use equipment for home-based training. Group A was considered the reference group of this RCT, as group B and C received additional digital interventions. The rationale for this RCT was to test which combination of strategies is more effective in increasing PA levels and maintaining PA levels in a long-term perspective of 18 months.

PAI is a personalized metric which takes into account age, sex, resting and maximum heart rate, and provides a score indicating whether the current PA level is sufficient to obtain or sustain good health (PAI score) (32). The goal setting is to reach and maintain a score of ≥100 weekly PAI. Individual feedback on the current PAI score was provided through the smartphone. Participants were also required to install and access regularly another app linked to the activity tracker (Zepp Life) so that the data stored in the PAI Health app were always up to date. Les Mills+ is a home-based online training solution offering videos of training classes (cardio, strength, flexibility, core etc.) available 24/7 trough a website, and is accessible from any device, smart TV, internet, PC, tablet or smartphone. The social support group was offered through a closed Facebook group aimed at providing a platform to share experiences, advice, support and motivation from peers. The Facebook group was administered by the project team, which regularly (one a week to twice a month) provided general information and educational advice about PA, motivational support, rewarding messages, technical and practical help.

Well-known external barriers to perform PA in inactive populations include environmental obstacles (e.g., weather), access to training facilities (due to distance or poor economic situation), time constrains, and lack of social support (8, 16). To decrease those barriers, we provided all participants with digital technologies and wearable devices at no cost for the participants. This was especially important to include inactive adults regardless of their socio-economic status (7). Home-based online training did not require more space than a few squared meters, making it feasible for most users. Online social support was based on a closed group on Facebook, a social media which is highly adopted and accessible by the target population of this study.

### Study design and data collection

Participants’ adoption, acceptability, and sustained use with the digital interventions were investigated through a mixed-methods approach, where different methods were selected, and their results supplemented each other to address the study objectives. We used triangulation as a technique to combine the qualitative and quantitative results, to gain a more complete picture of the study objective (33). Acceptability with the digital interventions was explored through the System Usability Scale (34), a standardized 10-item questionnaire with five response options per question (ranging from Strongly agree to Strongly disagree). Each of the interventions was evaluated through one questionnaire by the study participants who received it. The SUS was administered at 6-month follow-up. Adoption and sustained use were evaluated through a set of questions administered at 12-month follow-up (Supplementary Table 1). Study participants were asked whether they used the received intervention and for how long they used it. Depending on their answer, they were asked their opinion on the use or the main reasons not using it.

Semi-structured individual interviews were conducted via the video-based solution Teams (Microsoft, state, United States) at 6-month follow-up in a subsample of participants from all the three groups (*n* = 18) to collect in-depth information on adoption, acceptability, and sustained use. We sent an invitation by e-mail to a random sample of 50 participants asking whether they were willing to take part in an interview. We aimed to include the same number of participants in each group and achieve data saturation. The interview guide was structured around the following main themes: (1) motivation to participate in the project, (2) changes in physical activity habits and maintenance, (3) experience with the use of the three digital interventions, (4) perception of social support, and (5) experienced motivation and effects (Supplementary File 1). The interviews were performed by one researcher (USM) in the period January–June 2022 and lasted between 25 and 45 min. The interviews were audio recorded and then transcribed as a whole (verbatim transcription). Citations reported in this study were translated from Norwegian to English.

### Data analysis

The System Usability Scale (SUS) measures different aspects of a system; (1) Effectiveness (how users can achieve their objectives), (2) Efficiency (how much effort is expended in achieving the objectives), and (3) Satisfaction (was the experience satisfactory) (34). The following procedure was applied to calculate the score of SUS: The answer to each question ranging from strongly disagree (1) to strongly agree (5), is converted into a new score, ranging from 1 to 5. For odd-numbered questions (1, 3, 5, 7, and 9) the new score is the answer minus 1, for even numbered questions (2, 4, 6, 8, and 10), the new score is 5 minus the answer. This scales all values from 0 to 4 (with four being most positive response). The converted values are then summed, and the total multiplied by 2.5 (ref.). The final SUS score ranges from 0 to 100. Based on research, a SUS score above 68 is considered above average, and anything below 68 is below average (34).

Adoption of the three interventions was computed as the ratio between the number of respondents who used the intervention and the total number of respondents who answered the question. Sustained use was computed as the ratio between the number of respondents who still used an intervention after 1 year and all those who adopted the intervention. The qualitative data from the interviews were analyzed by a multidisciplinary team consisting of four members (USM, PZ, EHS and KA) using the Framework method (35, 36). Further, this approach identify commonalities and differences in qualitative data, before focusing on relations between different part of the data seeking to draw descriptive or explanatory conclusions around themes, and is especially used in multidisciplinary research teams. The research team had a background in health science, nursing, health technology and sports science. First, two of the transcribed interviews were randomly selected and read by the entire team to become familiar with the transcripts, develop initial impressions and ideas for codes. Second, the two transcripts were thoroughly read and independently analyzed by each member of the team. Interesting segments were underlined, and notes were made to describe the content of each passage with coding labels. Then the team met to share the coding labels previously assigned to the two transcripts. We analyzed every passage to discuss how it would be useful to address the research questions. The coding labels used to describe each passage were compared to find similarities in the interpretations of the content and to resolve differences. Further, a working analytical framework was developed around a set of codes that were explained by a short definition of their content. All the remaining transcripts were then assigned to the four members of the team and analyzed using the working analytical framework. New codes that arouse and that were not included in the initial framework were added to the already defined codes as additional impressions emerged. During this process, the team had regular meetings to discuss new codes, merge codes that were conceptually related and refine the initial analytical framework, until no new codes were generated. The quantitative results from the questionnaires and the qualitative findings from the interviews were finally analyzed and interpreted by triangulation, which is recommended in mixed methods research. This approach allows comparing multiple sources and consider where findings from each method agree (convergence), offer complementary information on the same issue (complementarity), or appear to contradict each other (discrepancy or dissonance) (33).

### Ethical considerations

The study is approved by the Regional Committee for Medical and Health Research Ethics (66573/REK nord) as well by the Data Protection Officer of the University Hospital of North Norway. The participants provided their written informed consent to participate in this study.

## Results

### Usability of the interventions

A total of 134 patients out of 183 answered the SUS at 6 months. The total score for the SUS was highest for the social support group (Table 1). The PAI smartphone application had a score of about 68 which is considered average, while the score for Les Mills + was under average. The results from the single questions provide a better understanding of the different dimensions of usability of the interventions (Supplementary Table 1). Overall, the Facebook group had higher scores regarding user friendliness and competence needed to use the intervention. However, PAI was the intervention that the participants intended to use most frequently.

**Table 1:** Scores for the System Usability Scale (SUS) questionnaires at 6 months, and SUS Item Scores (1–5) for the three digital interventions (*n* = 134).

Questionnaire	PAI	Online training	Online social support
SUS score, total, mean ± SD	**68.4 ± 22.4**	**61.4 ± 23.0**	**80.6 ± 15.7**
SUS score, each item			
1. I think that I would like to use this intervention frequently	3.4	2.8	2.9
2. I found this intervention unnecessarily complex	2.3	2.3	1.3
3. I thought this intervention was easy to use	3.6	3.2	4.4
4. I think that I would need assistance to be able to use this intervention	1.9	1.9	1.1
5. I found the various functions in this intervention were well integrated	3.1	3.0	3.5
6. I thought there was too much inconsistency in this intervention	2.1	2.3	1.7
7. I would imagine that most people would learn to use this intervention very quickly	3.7	3.5	4.5
8. I found this intervention very complicated to use	2.1	2.3	1.6
9. I felt very confident using this intervention	3.6	2.9	3.9
10. I needed to learn a lot of things before I could get going with this intervention	1.8	2.0	1.3

### Adoption and sustained use of the interventions

A total of 109 participants out of 183 answered the question administered at 12-month follow-up on whether they used the interventions they received. Adoption was highest for the PAI (89.9%) (Table 2). Adoption of online social support among respondents in group C was also above average (76.5%), while only one third of the respondents in group B and C reported to use home-based online training (31.4%). Most of those participants who adopted online social support were still using it after 12 months (96.2%). Sustained use for PAI after 12 months was lower (78.6%), but many of the participants used the interventions for more than 10 months. For online training most of the participants discontinued the use within 3 months (68.2%).

**Table 2:** Adoption and sustained use of the interventions at 12 months.

Measure	PAI	Online training	Online social support
Adoption	89.9%	31.4%	76.5%
Sustained use			
Still in use after 12 months	78.6%	18.2%	96.2%
Used it for 1–3 months	0.0%	68.2%	3.8%
Used it for 4–6 months	4.1%	4.5%	0.0%
Used it for 7–9 months	2.0%	4.5%	0.0%
Used it for 10–12 months	15.3%	4.5%	0.0%

### Qualitative interviews

Data saturation was achieved at a total of 18 interviews, with six participants included from each intervention group thirteen women and five men in the age range between 32 and 54 participated. The final analytical framework consisted of 26 codes grouped into five main themes: (1) Overall approach to physical activity, (2) Motivation, (3) Barriers to perform PA, (4) Effects of PA, (5) Usability and acceptability of the digital interventions (Table 3).

**Table 3:** Analytical framework.

Themes and codes	Description
*Overall approach to physical activity*	
Experience with physical activity*	Barriers for and/or experience with physical activity
Motivation/incentive to participate*	Incentive for participating in ONWARDS, barriers to physical activity
Experience with other digital interventions/devices*	If they have used other devices before and/or during study
Social support	Support from family, friends, colleagues etc.
*Motivation*	
Motivation from PAI	Feedback / rewarding from PAI
Demotivation from PAI	Technical issues hindering registration of PAI-points, types of exercise not rewarded etc.
Motivation from experienced effects	Response to training
Intrinsic motivation	Internal competition as motivation
Competition with others	Motivation training/competing with others (social)
*Internal barriers to physical activity*	
The “door step mile”	It feels prohibitive to “go out the door” for physical activity, hesitation
Oneself as a barrier	Want to be more active but do not have enough motivation/drive
Lack of “vitality”	Low vitality/energy to start exercise
Prioritization	Family, work, not prioritizing oneself
Health factors	Injury, illness, hindering physical activity
*External barriers to physical activity*	
Lockdown (pandemic)	Training centers closed, isolation, quarantine
Weather / season	Affects if you are physical active or not due to the seasons and/or weather
Where to exercise	Access to training center, nature, residence
Costs	As a barrier to perform physical activity (training center or training gear)
Life factors	Job, family, lack of time
*Effects*	
Impact on physical activity habits	Easier after joining ONWARDS, intensity, length, level, increased focus
Planning of physical activity	Goal setting, planning or not planning, (combination)
Health gains	Weight loss, better sleep, lower stress level, investing in own health
Well-being	Vitality, energy, bad conscience, self-esteem
*Experience with digital interventions*	
Experience with MiFit/PAI/wearable activity tracker	Included non-use/problems, experiences, user friendliness
Experience with Les Mills+	Included non-use/problems, experiences, user friendliness
Experience with Facebook group	Including non-use/problems, experience

*Before the study.

### Overall approach to physical activity (before the study)

The participants had different experiences and approaches to PA prior to the study. Some participants were used to exercise before (e.g., in their youth), but their PA level had decreased or completely stopped in their adult life, and they stated that they wanted to start doing PA again. Others considered themselves as physically active to some degree, such as going to work, walking with their dog, or hiking from time to time, but wanted to get in better shape by doing more regular exercise. Fewer participants had never or rarely been physically active before and wanted to start with participating in the ONWARDS study.

I used to be active earlier, but after having kids it has been difficult. I have always been exercising and enjoy it (ID 10).

Reasons for not being active were lack of motivation, lack of time, bad weather conditions, or restrictions during the pandemic. Others did not like to exercise. The lack of ability to plan and incorporate PA into the everyday life was also highlighted as a reason for not performing PA.

The participants had different views on how to increase their level of PA. While some just wanted to increase their number of daily steps, others wanted to perform regular training sessions (e.g., running, skiing, strength) several times a week. Many saw the ONWARDS study as an opportunity and motivation to be more active, exercise regularly, increase their fitness level and gain overview and control of their PA level.

I do not like to be physically active; it is not integrated in my everyday life although I know it is important (…). So, I thought “why not”? Maybe participating in this project can help me to get a kick-start (ID 11).

Social support from family, friends, or colleagues to be physically active varied between the participants. While it was perceived as important for some, others did not experience any need for social support. Some had previous experience with wearable devices for PA (e.g., sport watches measuring heart rate, number of steps, sleep quality) or applications (e.g., for weight control), but none had used PAI or monitored their level of PA over a long period of time.

### Motivation and demotivation

A major motivational factor for many of the participants was the feedback on their individual PAI score and the earning system in the wearable activity tracker with the connected PAI app. The reward from an increasing PAI score was perceived as a boost to be physically active, giving a good feeling of mastery when achieving the expected goal (100 PAI/week). The feedback when the PAI score was low motivated several participants to increase their PA level. Over time, many participants learned how much extra effort they needed to do to obtain 100 PAI/week.

When I have reached a high PAI score, I get motivated to maintain the score. I get kind of attached to the thing (PAI), checking the PAI score in the morning to find out how much effort I need to do to maintain the PAI level that day (ID 16).

Several participants got motivated from the feeling of physical and psychological well-being they gained by being more physically active. In addition, a strong motivational factor was being able to maintain this feeling of well-being, confidence, and ability to make PA as a routine in their everyday life. For some participants, motivation to continue with PA lasted over time and some experienced that it was easier to increase the level of PA even if the PAI score had been low for a period. At the same time, the feedback of their PAI score felt demotivating for others, especially if they for different reasons did not have the opportunity to be physically active for a period, and the PAI score reminders were perceived as annoying or stressful.

I am receiving fewer points than I deserve. I am probably very concerned about the number of PAI-points. But at the same time, I think it gives me a motivation to work out on days where I normally would not work out (ID 3).

The lack of PAI score from low-intensity activities like strength training was also perceived as a demotivation for some, as they had been active but were not able to reach the expected PAI goal. Some did not see any difference at all in their motivation to be more PA and expressed the main reason to be the lack of intrinsic motivation rather than the digital devices they used. Being active together with family members, friends or colleagues was an important motivation to increase PA for some, while others were motivated from having some time for themselves.

### Barriers to physical activity

Internal barriers are factors within the persons themselves hindering them from being physically active. The *doorstep mile* was often named and explained as *it feels prohibitive to go out the door for physical activity, making the person hesitating and stay indoors doing nothing instead.* Low vitality, as well as illness or injuries, were also common barriers to be more physically active. Many participants described that it was hard to prioritize time to be PA, as family and work often came first.

The total amount of things to do and the energy one uses is not always enough for both work, family, and me. The things that most often are deprioritized are those related to me. It is very wrong, then you have less to give to everyone else also. But that is what happens, and partly still do. When you have done everything else that day, and there is no time for me, there is nothing (energy) left (ID 5).

Several external barriers for PA were pointed out, one of them being the pandemic situation with isolation, quarantine and training centers closed for several weeks or months. Lack of space or gear to exercise at home was named as a barrier, although most participants preferred to be outside for PA. Others did not have access to training centers due to long distances, living in rural areas or the cost of a membership was perceived as too high. Poor weather conditions, especiallyin winter, affected some participants, hindering people to be outside and be active for parts of the season. The period with no sun (the dark period) in Northern Norway lasting from November to January was a common barrier for some.

Now we have recently had the dark period. It is often a heavy period up here. It makes me more passive. It has been colder as usual, so I have not done so much winter activities as usual. But now the sun is back, it is getting a bit warmer and easier (ID 5).

Some experienced their work situation, characterized by responsibility, traveling or long working hours, as a barrier to perform as much PA as they wanted and needed.

### Experienced effects and behavior change

Most of the participants experienced that participating in the study had a positive impact on their PA habits, describing that they became more focused on performing PA and that it felt easier to be more physically active in their everyday life than earlier. Some explained they found a lot of joy and fun in performing PA now, and they got in better shape.

It really affects me, It’s really fun and a lot more motivating when I see that I can run four times a week, and I am in a much better shape now (ID 20).

Some participants set goals for their activities/training, doing regularly exercise two or more times a week on fixed days, or had a plan for the different types of exercise during a week. Others were happy just to go out for a walk sometimes during the week, and were more focused on doing everyday activities (i.e., walking instead of taking the bus). Many participants made a plan to have time to prioritize PA, whilst others were active when it was possible (e.g., while their children were on activities, on their way to and from work). Some experienced that they lost some weight as a result of being more active, with a consequent positive effect on their self-esteem and ability to perform PA.

It is good to be physically active and feel the endorphins I gain after being active. It is a satisfactory feeling to be happy with myself and not have the bad conscience for it (ID 3).

Some participants experienced better sleep quality or lowered stress levels when doing PA regularly. Others pointed out the effects of their increased activity level as a good and important overall investment in their own health, especially for those being from the higher age group (≥40). There were also participants who had not experienced any objective or subjective health gains during the first 6 months of the study.

I had a desire of increasing my level of PA by joining this study, but nothing has changed, and I have a persistent low level of PA. It is not exactly motivating, and I do not feel any benefit of the interventions (ID 12).

### Usability and acceptability of the digital interventions

Experiences from using the activity tracker Mi Smart Band varied among participants. Some thought that the activity tracker was too simple and small, especially for those who previously had used other sport watches (Garmin, Polar, Apple). In a few cases the device needed to be replaced with a new one after being used 24/7 for several months.

It has gone perfectly well. It takes some time to get used to it (the Mi Smart Band), that’s the only thing, Otherwise, it is very nice and easy to use. Although it lacks some activities you can choose on the watch. So far, it’s pretty good (ID 17).

Technical issues were experienced by some, especially with synchronizing the activity tracker with the two apps (PAI Health and Zepp Life), which could make participants demotivated to continue in the study because their fitness scores were not up to date.

I am using the apps in between just to update and synchronize, and to follow my PAI score (…). But I do not use it actively, I think the system seems kind of cumbersome (ID14).

Most participants liked to follow their PAI score and found it very motivating to reach the goal, or notice they need some more PA that day or week to reach 100 PAI-points.

Few participants used Les Mills+. Most participants perceived the website and classes as too advanced, with too many options and difficult to navigate to find a suitable training program fitting their personal level of fitness. Lack of space or equipment at home was also an issue for some. Some perceived that online training did not suit them or did not increase their motivation, others preferred to exercise outside or at their local gym instead of using online home-based exercise. Only few participants had no problem using Les Mills+ and found the site easy to use, but only one used it regularly.

I think I only tried it twice, but I did not feel that it gave me anything. That Les Mills-thing was not for me (…). I tried an activity for the whole body, but it was too much jumping up and down. It is not that I do not like group training, but on a digital platform is not for me. I like a real-life instructor present (ID 12).

Most participants did not find the Facebook group motivating or to have any influence on their PA level. Some participants did not join the group at all because they were not on Facebook, whilst others followed the group but did not actively interact. Another reason for not being active in the Facebook group was the fear of losing their own feeling of mastery when comparing themselves to other group members. Some also recognized another participant in the group, making them even more reluctant to post from their own activities or sharing experiences. Caution in posting things and pictures about themselves was highlighted as a factor for not being active in the group, although some participants liked status and pictures posted on PA from other participants. Some wanted more information and follow-up from the project group, like posts on how many PAI scores during a week or month they had, or other information regarding motivation to perform PA.

That Facebook group …there has not been so much activity there, but it is fun to see when someone post pictures. I do not think it has motivated me to do something about my own activity level (ID 20).

## Discussion

This mixed method study explored inactive adults’ adoption, acceptability and sustained use of three different digital interventions aimed at increasing PA. The activity tracker with PAI was perceived as the most successful of the three digital interventions due to its sufficient usability, it was widely adopted and used over time due to its perceived effect on motivation and increase in PA levels. Although acceptability and sustained use of online social support were high, the intervention was not perceived as motivational for PA. Online training was the intervention with the lowest adoption, usability and sustained use. Personalized feedback and goal setting through PAI seemed to be major factors for a positive behavioral change among the study participants. These behavioral change components were not included in the two other interventions.

Personalized feedback from digital devices, like smartphone apps, is considered as an important facilitator for lifestyle behavior change (24) and an effective component to increase motivation for PA (10, 24, 37). At the same time, other studies showed inconsistent results on the how personalized feedback affects motivation for PA (20, 22). Goal setting is another component which is frequently included in PA interventions, as well as in technical devices like fitness/activity trackers to increase motivation for users to be more active (38). A scoping review suggested that assigning a goal to users is more effective than letting the users set their own goal (18). A recent study also reported that users preferred goals matching their abilities, which made it more possible to boost motivation to continue regular PA (22). An advantage of using PAI compared to other activity trackers (e.g., pedometer trackers) is that PAI is personalized to a person’s age, gender and fitness level, and provides a single individual score indicating whether the current PA level is sufficient to obtain or sustain good health (39, 40). Most of the study participants experienced the PAI score and goal of 100 PAI/week as major factors to gain motivation and positive effects on physical and mental well-being. These, in turn, contributed to a higher adoption (89.9%) and sustained use (78.6%) of this intervention at 12 months compared to home-based online training and online social support. At the same time, a disadvantage of using PAI for goal setting on PA is the reliance on the users’ heart rate patterns during PA (32), which may favor aerobic activities over resistance-based activities (e.g., strength exercise). As a consequence, some participants found the feedback on PAI score to be demotivating or stressful, especially in periods of low activity or when receiving much fewer PAI points for strength activities compared to cardiovascular activities.

We expected home-based online training and online social support to give an additional effect to the users. However, as these two interventions were delivered as an addition to PAI, which was perceived as the most effective and useful intervention, their added impact and effect was limited. If home-based online training and online social support were delivered as stand-alone interventions, the impact on motivation and experienced effect may have been higher.

This study was partly conducted during the COVID-19 lockdown or when socializing with others was limited, and it was expected that home-based online training would provide a good alternative to do PA in this time of limited PA resources. A study on the acceptability of online workout classes by a population in isolation during the pandemic reported good feedback on the impact on PA (10), whereas other found unclear results of online workout classes in young adults and the impact of social network to promote PA (12, 28). Although the participants in this study live in an area of Norway often affected by harsh weather conditions, most of them preferred exercising outdoors. This may have affected the perceived usefulness of home-based online training. While the usability score was acceptable (moderate), only few participants used the intervention for more than a short period of time, showing low adoption and sustained use. Other studies on web-based video-tailored PA interventions report short adherence to the intervention and a low perceived usefulness from the users (27). Another reason which might have affected acceptability, adoption and sustained use was that the website for online training was too overwhelming and there were too many classes to choose from.

The purpose of the online social support was to offer a closed online forum where participants could exchange experiences, discuss and motivate each other to perform PA, reach PAI point goals and create a social support environment. Although the intervention had the highest usability score and a good score on adoption and sustained use, was perceived to have a limited additional value relative to PAI on the motivation to do PA. Studies on the impact of peer-support interventions to promote PA through social media showed inconsistent results. One study reported that more activity and feedback from the users in a social support group on Facebook increased the participants PA level slightly (30). Another study reported the effect of social support through Facebook groups on PA, but only in the short-term (12). A recent study reported that adding goal setting, self-monitoring, tailored feedback and educational information was perceived as the most effective strategy for increasing PA in digital social network groups (15). Interestingly, the usability of the online social support in the current study was perceived as very high. Facebook is a well-known social network platform, and many of the users were familiar with it prior to the study. Furthermore, its wide use beyond the purpose of this study might have contributed to the increased sustained use observed. This indicates that established social networking platforms with widespread use can be ideal delivery media for digital interventions.

Digital interventions for increasing PA have shown positive effects mostly in younger populations (13, 15, 28). The current study targeted a population of mostly middle-aged (30–55 years old) inactive adults, who were presumably at risk of developing lifestyle diseases caused by the lack of PA. Moreover, most studies have a short-time duration, making it difficult to measure long-term adoption and sustained use of digital interventions (17, 41). The current study was characterized by a unique duration of 18 months, and most of the participants were still using PAI after 1 year. The high sustained use of this intervention shows the potential for long-term adoption for inactive adults.

### Strengths and limitations

The study was designed without an ordinary control group. All participants were provided with an activity tracker and PAI, and home-based online training and online social support were additional interventions in two groups. The design of this study might have limited the potential effect of these interventions compared to PAI. A mixed-method approach is an advantage as the study was based on both quantitative data from a larger population as well as qualitative data from a selected group of users. Although the information gained from the scores on acceptability, adoption and sustained use, the in-depth experiences reported from the qualitative interviews allowed to further examine why and how participants perceived the different digital interventions. The framework approach to qualitative analysis was chosen as it is a flexible tool that can be adapted for use in multidisciplinary research teams and within research projects using a mixed-method design (35). The quantitative data were based on self-reported data after 6 and 12 months. Self-reported data are known to be a risk for bias compared to objective measurements.

## Conclusion

This study evaluated the adoption, acceptability and sustained use of three different digital interventions aimed at promoting physical activity in inactive adults. The activity tracker with the personalized metric PAI on a mobile app was the most successful intervention, with a satisfactory usability and perceived positive effects on motivation and behavior change which, in turn, contributed to a high adoption and sustained use. The online social support had a high acceptability and sustained use among the study participants, but the intervention was not perceived as particularly motivational to increase PA. Online training had low adoption, usability and sustained use. Personalized digital interventions integrating behavior change techniques such as individual feedback and goal setting are more likely to support motivation for behavioral change in inactive adults and increase acceptability, adoption and sustained use. Future studies should investigate which digital interventions or combinations of different interventions are more successful in promoting PA among inactive adults according to the characteristics and preferences of the users.

## Data availability statement

The original contributions presented in the study are included in the article/Supplementary material, further inquiries can be directed to the corresponding author.

## Author contributions

UM: Formal analysis, Investigation, Methodology, Writing – original draft, Writing – review & editing. ES: Formal analysis, Investigation, Writing – review & editing. KA: Formal analysis, Investigation, Writing – review & editing. PZ: Formal analysis, Investigation, Methodology, Project administration, Writing – review & editing.
