# Supplementary material for: Adoption, acceptability and sustained use of digital interventions to promote physical activity among inactive adults: a mixed-method study
Source: Front Public Health. 2024 Jan 4;11:1297844. doi: 10.3389/fpubh.2023.1297844 (PMC10794730; doi:10.3389/fpubh.2023.1297844)
Supplement: Supplementary file 1 [file Data_Sheet_1.docx]

**Interview guide ONWARDS**

Information to participants before starting the interview:

“This interview is about your experiences related to participating in the ONWARDS project. The topics we will cover are broadly: how you experience being physically active, what motivates/demotivates you, perceived obstacles for being active, your experience with the use of digital tools and social support. The interview will be recorded on an audio recorder and then printed before analysis of the data. All personal and identifiable information about you will be removed and it will not be possible to identify you in the further process after this interview."

***Themes and key questions:***

1. **Motivation to participate**

Can you tell me what motivated you to participate in ONWARDS?

1. **Changes in physical activity habits and maintenance**

Have your physical activity habits changed since you started in ONWARDS? If yes, please elaborate

Is there anything preventing you from being active now? If yes, please elaborate

Do you fell it easier to be physically active now than before ONWARDS? If yes, can you elaborate on why? (keywords: motivation, mastery, obstacles)

1. **Use of digital interventions**

Did you use digital tools for physical activity before joining ONWARDS? If so, what type and how often?

Can you tell me which one of the digital tools in ONWARDS you use today?

Which one do you perceive as most useful to you and why?

How is your experience with the use of PAI/LesMIlls+/Facebook group on your motivation to be physically active?

How do you experience the user-friendliness of the different digital tools?

How does the digital tools fit you and your preferences?

1. **Social support**

Can you tell me how you experience support from family, firneds, colleagues etc. regarding being physically active?

How do you plan or schedule time for being active?

1. **Experienced motivation and effects**

Have you experienced any changes (physical, psychological, social) after participating in ONWARDS?

How are your experiences with maintaining physical activity since you started? (keywords: motivation, external factors)
