# Supplementary material for: Adoption, acceptability and sustained use of digital interventions to promote physical activity among inactive adults: a mixed-method study
Source: Front Public Health. 2024 Jan 4;11:1297844. doi: 10.3389/fpubh.2023.1297844 (PMC10794730; doi:10.3389/fpubh.2023.1297844)
Supplement: Supplementary file 2 [file Table_2.DOCX]

**Supplementary table 1.** Additional questions at 12 months on adoption and sustained use of the three digital interventions.

| Questions* | Answers |
| --- | --- |
| Have you used the intervention? | Yes/no |
| If yes, for how long have you used the intervention? | \| 1= I am still using it \| \| --- \| \| 2= I used it for 1-3 months \| \| 3= I used it for 4-6 months \| \| 4= I used it for 7-9 months  5= I used it for 10-12 months \| \|  \| |
| How do you feel about using the intervention? | Free-text |
| If no, what is the reason for not using the intervention? | Free-text |

*****All 4 questions were asked for each of the three digital interventions (total 12 questions)
